# A natural product-like JAK2/STAT3 inhibitor induces apoptosis of malignant melanoma cells

**DOI:** 10.1371/journal.pone.0177123

**Published:** 2017-06-01

**Authors:** Ke-Jia Wu, Jie-Min Huang, Hai-Jing Zhong, Zhen-Zhen Dong, Kasipandi Vellaisamy, Jin-Jian Lu, Xiu-Ping Chen, Pauline Chiu, Daniel W. J. Kwong, Quan-Bin Han, Dik-Lung Ma, Chung-Hang Leung

**Affiliations:** 1 State Key Laboratory of Quality Research in Chinese Medicine, Institute of Chinese Medical Sciences, University of Macau, Macao, China; 2 Department of Chemistry, Hong Kong Baptist University, Kowloon Tong, Hong Kong, China; 3 Department of Chemistry, The University of Hong Kong, Hong Kong, China; 4 The State Key Laboratory of Synthetic Chemistry, The University of Hong Kong, Hong Kong, China; 5 School of Chinese Medicine, Hong Kong Baptist University, Kowloon Tong, Hong Kong, China; Duke University School of Medicine, UNITED STATES

## Abstract

The JAK2/STAT3 signaling pathway plays a critical role in tumorigenesis, and has been suggested as a potential molecular target for anti-melanoma therapeutics. However, few JAK2 inhibitors were being tested for melanoma therapy. In this study, eight amentoflavone analogues were evaluated for their activity against human malignant melanoma cells. The most potent analogue, compound **1**, inhibited the phosphorylation of JAK2 and STAT3 in human melanoma cells, but had no discernible effect on total JAK2 and STAT3 levels. A cellular thermal shift assay was performed to identify that JAK2 is engaged by **1** in cell lysates. Moreover, compound **1** showed higher antiproliferative activity against human melanoma A375 cells compared to a panel of cancer and normal cell lines. Compound **1** also activated caspase-3 and cleaved PARP, which are markers of apoptosis, and suppressed the anti-apoptotic Bcl-2 level. Finally, compound **1** induced apoptosis in 80% of treated melanoma cells. To our knowledge, compound **1** is the first amentoflavone-based JAK2 inhibitor to be investigated for use as an anti-melanoma agent.

## Introduction

The incidence of melanoma has increased over the past three decades [1,2], and its mortality rate is higher than another cancers [3,4]. However, less special drug for metastatic melanoma is approved for the first-line therapy [5–9].The Janus kinase 2 (JAK2)/signal transducer and activator of transcription 3 (STAT3) pathway is overactivated in many human cancers, including melanoma [10,11]. Therefore, inhibiting JAK2 is a potential anticancer strategy. AG490, the first JAK2 inhibitor, selectively blocks cell growth *in vitro* and *in vivo* by inhibition of JAK2 activity and inducing apoptosis [12]. Another potent JAK2 inhibitor, NVP-BBT594, suppressed activation loop phosphorylation of JAK2 [13]. To the best of our knowledge, just a small number of JAK2 inhibitors were being tested for cancer therapy on the status phase Ⅰ or phase Ⅱ, and only one JAK2 inhibitor, Ruxolitinib (INC424), was approved by Food and Drug Administration (FDA) [14]. Within the realm of natural products or natural product analogues, cucurbitacin Ι and BBMD3 have been reported to inhibit the JAK2/STAT3 pathway [10,15].

Traditional Chinese herbal medicines provide a rich resource of bioactive structure for pharmaceutical drug development [16–36]. Flavonoids are natural polyphenolic substances that are widely found in Traditional Chinese medicine [37] and have been investigated as potential anti-cancer agents [38]. Previously, our group reported that the activities of amentoflavone and its analogues on JAK2 kinase against human erytholeukemia cells (HEL). The biflavonoid amentoflavone was identified as an inhibitor of JAK2 activity using a structure-based virtual screening approach, which showed promising anticancer activity against HEL cells [34]. However, the efficacy of amentoflavone analogues against malignant melanoma, a widespread and deadly cancer, has not yet been investigated. Therefore, in this study, eight amentoflavone analogues were evaluated for their anticancer activities against human melanoma cells. Our findings demonstrate that the amentoflavone analogue compound **1** is a potent inhibitor of the JAK2/STAT3 signaling pathway against melanoma cells, suggesting that this natural product scaffold could deserve further attention for the development of anti-melanoma therapeutics.

## Materials and methods

### Reagents

All antibodies were purchased from Cell signaling Technology. All compounds were dissolved in dimethyl sulfoxide (DMSO) at a stock concentration of 10 mM. Human malignant melanoma (A375) cells, human malignant melanoma (A2058) cells, human prostate cancer (PC3) cells, human prostate cancer (DU145) cells, and human liver cancer (HepG2) cells were obtained from American Type Culture Collection. The hepatocyte cell line LO2 was obtained from Chinese Academy of Science, Cell Biology of Shanghai Institute, Shanghai, China.

### Cell culture

A375, A2058, PC3, DU145, HepG2 and LO2 cells were cultured at cell density of 4–5 × 10^5^ cells/mL in DMEM (Dulbecco’s Modified Eagle Medium) with high glucose and L-glutamine and supplemented with 10% fetal bovine serum (FBS) and 1% penicillin (100 units/mL)/streptomycin (100 μg/mL) at 37°C and 5% CO_2_.

### Cell viability assay

A MTT assay was used to evaluate the antiproliferative effect of the natural products. Cells were seeded at a density of 5,000–6,000 cells per well in 96-well plates. When the density of the cells reached 50% confluence, the cells were treated with compounds at final concentrations ranging from 0.01 to 10 μM for 48 h. MTT was added to each well at a final concentration of 1 mg/mL for a further 4 h. After removing the medium from the cells, 100 μL DMSO was added to each well. The viability of the cells was measured by recording the absorbance of each well at 490 nm using a SpectraMax M5 microplate reader after shaking the plate for 10 min at room temperature in the dark.

### Western blotting analysis

A375 cells were treated with vehicle, compound **1** or NVP-BBT594 for 24 h, and then harvested and washed twice with ice-cold PBS. Protein samples were extracted with radio-immunoprecipitation assay buffer (RIPA) lysis buffer containing 1% cocktail and 1% PMSF. 30 μg of total proteins were resolved on an SDS/PAGE gel and transferred to a polvinylidene fluoride (PVDF) membrane. Blots were blocked in 5% none-fat dry milk with TBS containing 0.1% Tween-20 for 1 h and probed with primary antibodies to JAK2, p-JAK2, STAT3, p-STAT3, caspase-3, PARP, Bcl-2, HIF1α, ubiquitin or β-actin and GAPDH with gentle agitation overnight at 4°C. Then, membranes were washed five time with TBST. After incubation with secondary antibody for 1 h. Signals of proteins bands were detected using enhanced chemiluminescent Plus reagents (GE Healthcare) and analyzed by Image Lab.

### Cellular thermal shift assay

Cellular thermal shift assay was performed to evaluate the target engagement of compound **1** in A375 cells. Briefly, after collecting A375 cell lysates, the cells lysates was separated in the same aliquots. Each aliquot was treated with **1** (1 μM) or DMSO. After incubation at room temperature for 30 min, the complex-treated lysates were divided into 50 μL in each of PCR tubes and heated individually at different temperatures (Veriti thermal cycler, Applied Biosystems/Life Technologies). The heated lysates were centrifuged and the supernatants were analyzed by SDS-PAGE followed by western-blot assay by probing with anti-JAK2 antibody.

### Apoptosis assay

To evaluate apoptosis induction, a FITC-Annexin apoptosis detection kit (BD Biosciences, San Jose, CA, USA) was utilized. The experiment was performed according to the manufacturer’s instructions. Briefly, A375 cells were seeded at density of 2.5 × 10^5^ in a 6-well plate, and were treated with compound **1** at different concentrations (0.03 to 3 μM) for 24 h. Cells were harvested and washed twice with ice-cold PBS, and then resuspended in 1 × binding buffer followed by incubation with Annexin V/PI solution for 15 min at room temperature. The samples were immediately analyzed by flow cytometry using a C6 Flow Cytometer™ system (BD Biosciences). At least 2 × 10^5^ cells were analyzed for each sample.

### Statistical analysis

Student’s t-test was performed using SPSS 12.0 software. The results are presented as the mean±SD. **p<0.01 was considered to be significantly different.

## Results

### Identification of 1 as a potent inhibitor of human melanoma cell viability

**Fig 1:**
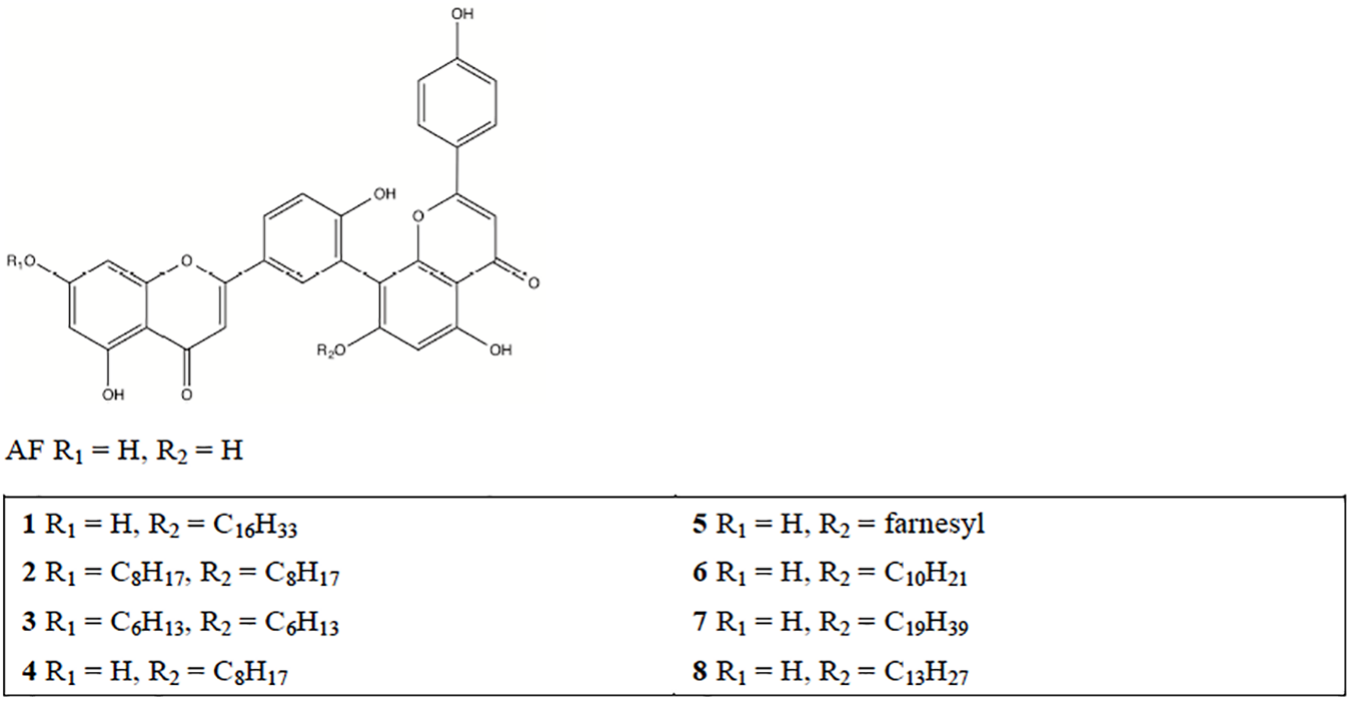
Chemical structures of amentoflavone (AF) and analogues 1–8.

**Fig 2:**
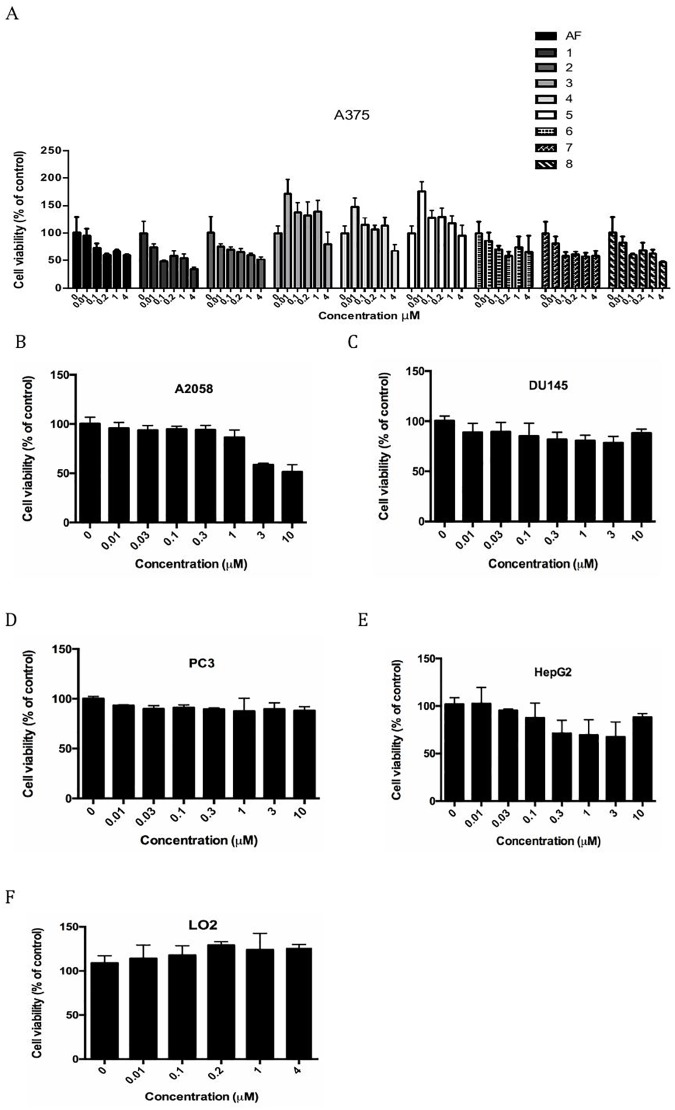
Effects of compounds on cell viability as determined by an MTT assay. A375 cells were treated with 0.01 to 4 μM of compounds or amentoflavone for 48 h. LO2 cells were treated with the same concentration of compound **1** for 48 h. PC3, DU145, HepG2 and A2058 cells were treated with 0.01 to 10 μM of compound **1** for 48 h. Error bars represent the standard deviations of results obtained from three independent experiments.

Eight amentoflavone analogues (Fig 1) were synthesized previously [34]. For primary screening, the MTT assay was performed to assess the ability of the analogues to inhibit the proliferation of A375 cells. Amentoflavone was used as the positive control. The results showed that compound **1** showed potent anticancer activity against the A375 cell line, with an IC_50_ value of 0.37 μM, compared to other 0.50 μM for amentoflavone (Fig 2A). In addition, compound **1** showed relatively non-toxic towards DU145, PC3, HepG2, A2058 and LO2 cells (IC_50_ ≥ 10 μM) (Fig 2B–2F). These data suggest that compound **1** could be regarded as a potential chemotherapeutic agent against human melanoma.

### Effect of compound 1 on the JAK2/STAT3 pathway

**Fig 3:**
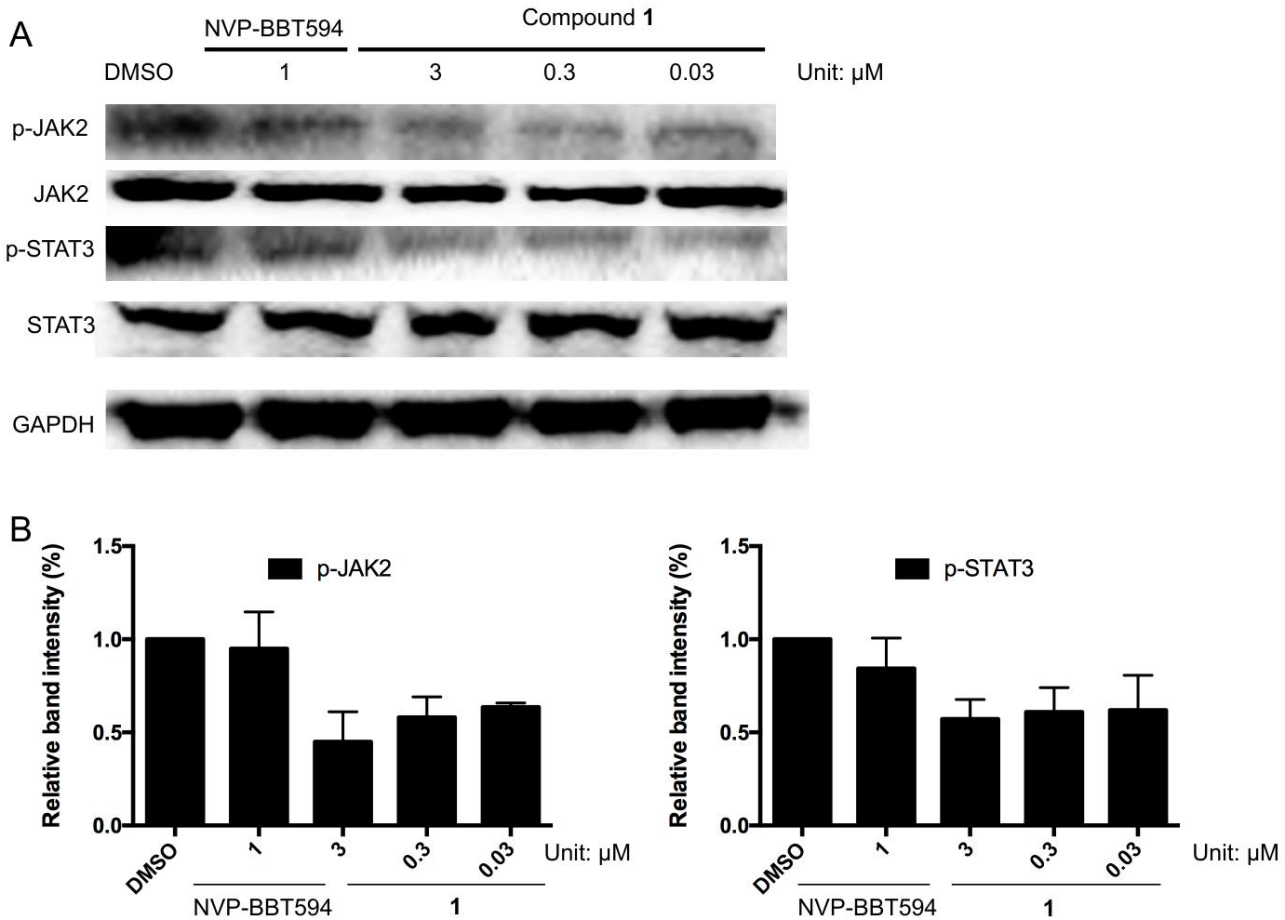
Compound 1 reduces phosphorylation of JAK2, STAT3 in A375 cells. A375 cells were treated with the indicated concentrations of compound **1** (0.03 to 3 μM) or positive control compound NVP-BBT594 (1 μM) for 24 h. Protein lysates were analyzed by Western blotting with the indicated antibodies. Error bars represent the standard deviation of triplicate results.

In order to determine whether compound **1** inhibits the JAK2/STAT3 pathway *in cellulo*, Western blotting assays were performed. A375 cells were treated with compound **1** (0.03 to 3 μM) for 24 h before measurement. The results showed that compound **1** suppressed the phosphorylation of JAK2 and STAT3, but had no discernible effect on total JAK2 and STAT3 levels (Fig 3).

### Cellular thermal shift assay

**Fig 4:**
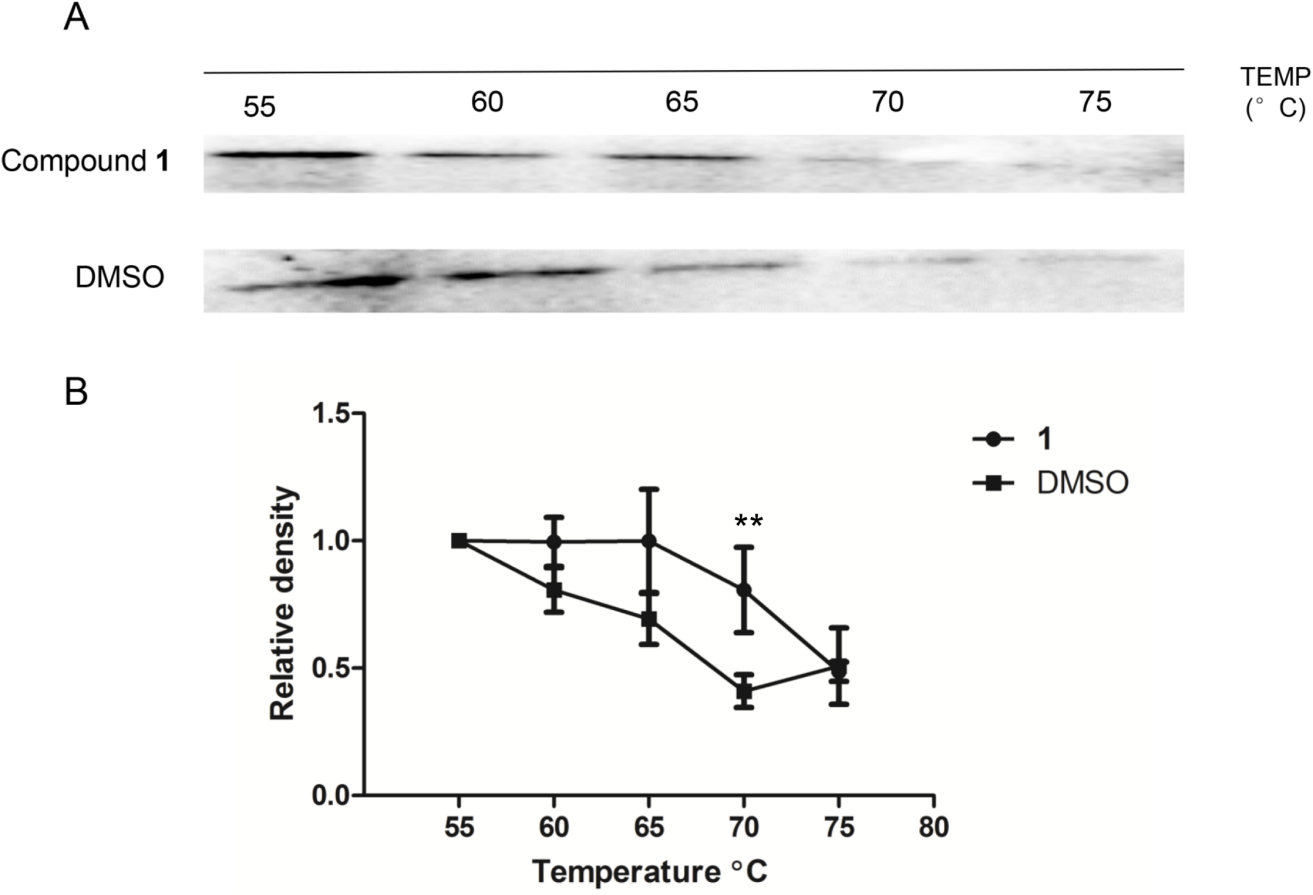
Compound 1 stabilizes JAK2 in A375 cells. **(A)** Stabilization of JAK2 by **1**. **(B)** The band intensity of JAK2 with different temperatures. The data were normalized to the JAK2 level of control group at 55°C and are expressed as the means ± SD of three individual experiments. The data were analyzed using Image Lab. Error bars represent the standard deviation of triplicate results. Significant differences versus control at the same temperature are indicated by **p < 0.01.

To evaluate whether JAK2 is engaged by **1** in cell lysates, a cellular thermal shift assay (CETSA) was applied [39]. The JAK2 content in the soluble fraction was determined by Western blotting. The results showed that **1** could stabilize JAK2 in treated cell lysates, as indicated by an obvious shift of the melting temperature of JAK2 content in the soluble fraction (Fig 4A and 4B). This result indicates that the thermal stabilization of JAK2 is engaged by **1**.

### Compound 1 induces cell apoptosis

**Fig 5:**
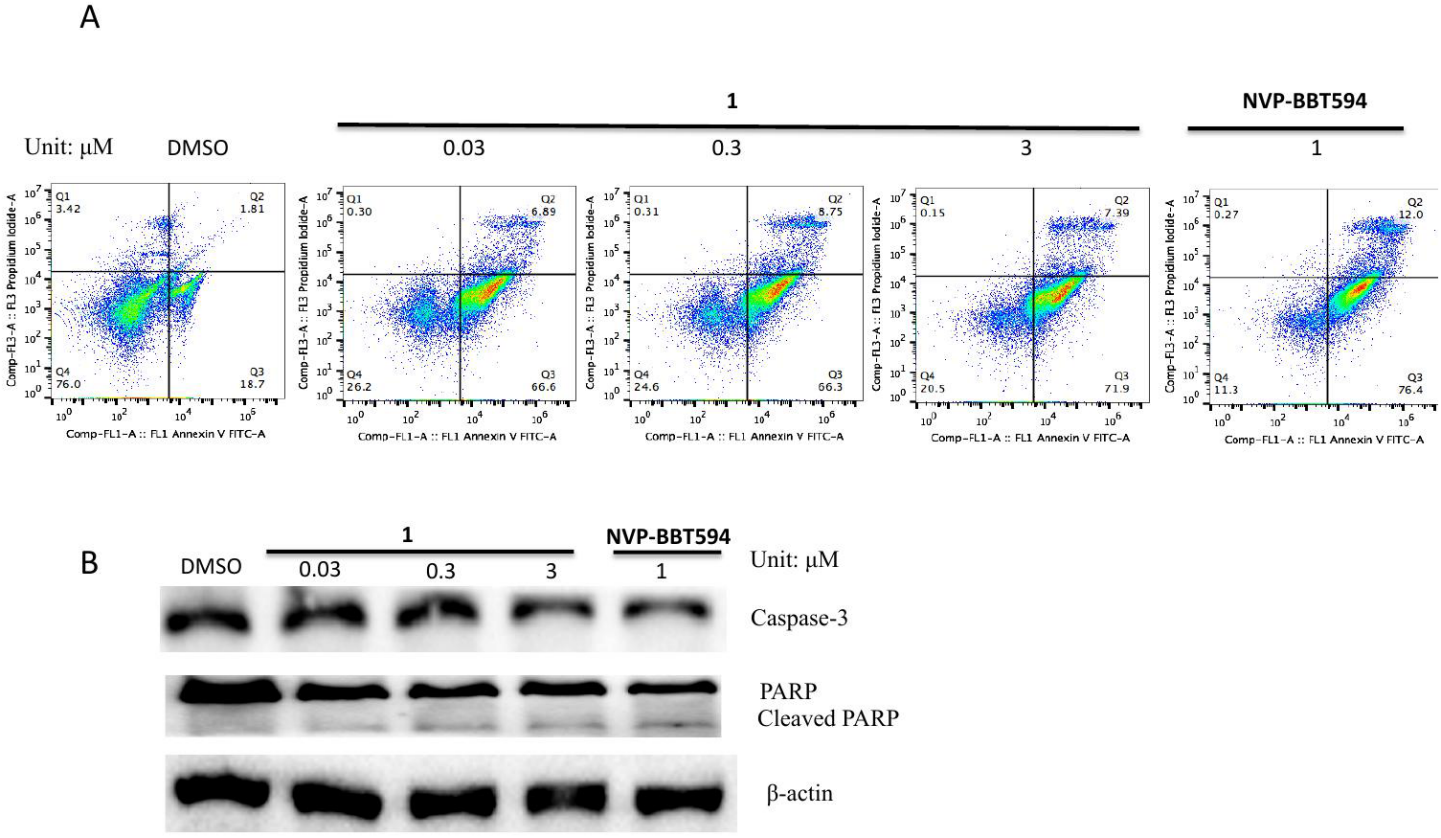
Compound 1 induces apoptosis in A375 cells. A375 cells were treated with the indicated concentrations of compound **1** (0.03 to 3 μM) or positive control compound NVP-BBT594 (1 μM) for 24 h. **(A)** A375 cells were stained with PI and Annexin V, and were analyzed by flow cytometry. **(B)** Protein lysates were analyzed by Western blotting with the indicated antibodies.

**Fig 6:**
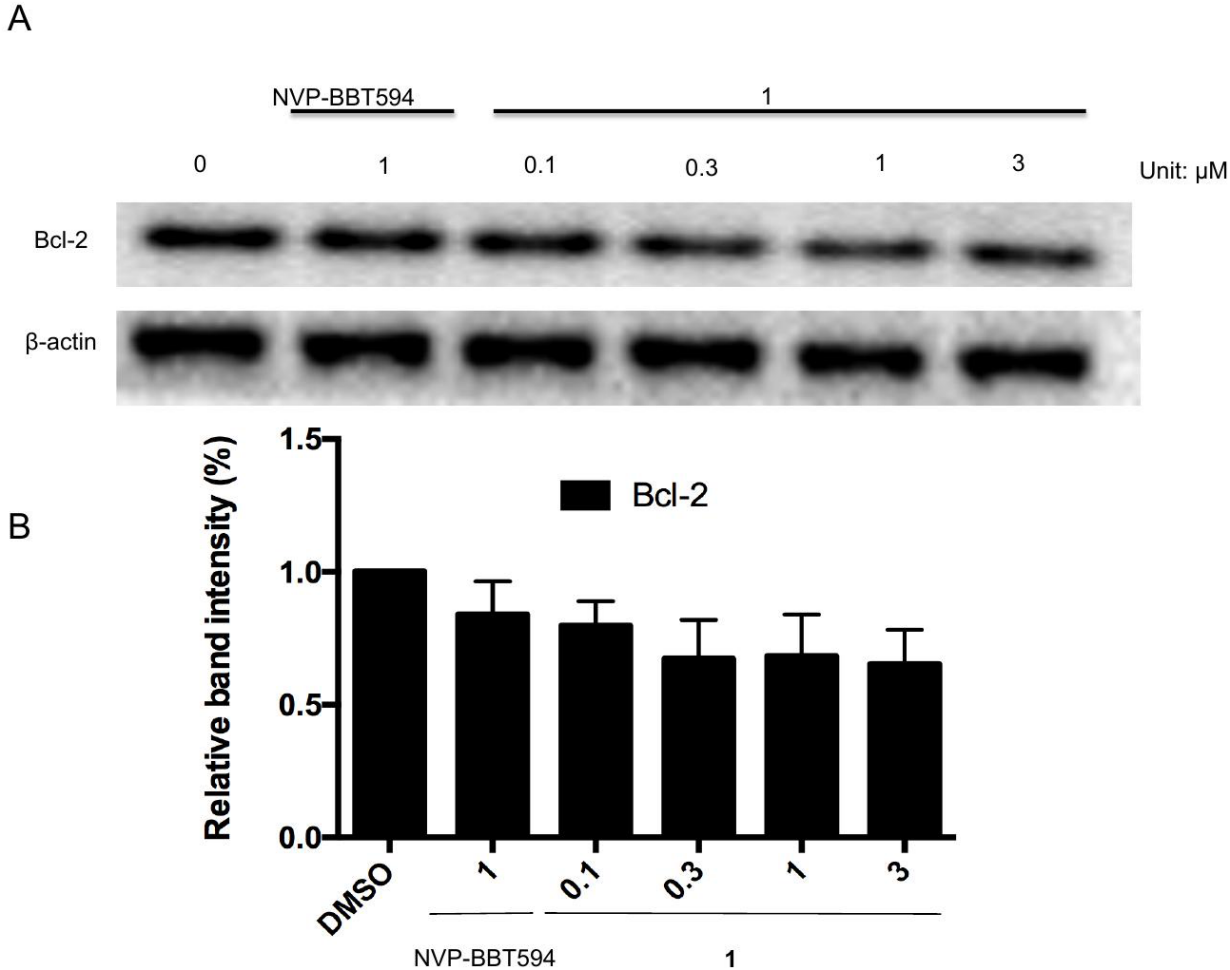
Effect of compound 1 on Bcl-2 level in A375 cells. **(A)** A375 cells were treated with the indicated concentrations of compound **1** (0.1 to 3 μM) or positive control compound NVP-BBT594 (1 μM) for 24 h. Protein lysates were analyzed by Western blotting with the indicated antibodies. **(B)** The data were analyzed using Image Lab. Error bars represent the standard deviation of triplicate results.

**Fig 7:**
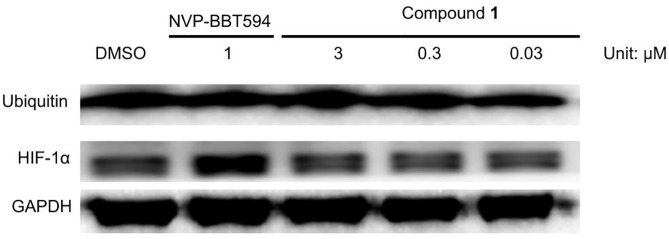
Effect of 1 on HIF1α and ubiquitin in A375 cells. A375 cells were treated with the indicated concentrations of compound **1** (0.03 to 3 μM) or positive control compound NVP-BBT594 (1 μM) for 24 h. Protein lysates were analysed by Western blotting with the indicated antibodies.

Activation of STAT3 can suppress apoptosis through inhibition of activated caspase-3 [40]. The cleavage of PARP and activation of caspase-3 are widely regarded as markers of apoptosis [40]. To confirm whether compound **1** induces apoptosis, A375 cells were exposed to 0.03 to 3 μM of compound **1** for 24 h, and the level of apoptosis was assessed by flow cytometry using annexin V and PI staining. The results showed that compound **1** could induce apoptosis in approximately 80% of the cells (Fig 5A). Moreover, compound **1** induced the cleavage of PARP and decreased caspase-3 levels (Fig 5B). Stat3 is known to regulate the expression of various anti-apoptotic and pro-apoptotic proteins [40]. Our results showed that compound **1** suppressed Bcl-2, an anti-apoptotic protein that is upregulated by STAT3 (Fig 6A and 6B). Taken together, these results indicate that **1** could induce apoptosis in A375 cells, presumably as a result of its effects on PARP, caspase-3 and Bcl-2 that are under the control of the JAK2/STAT3 signaling pathway. Additionally, compound **1** had no effect on HIF1α and ubiquitin levels (Fig 7).

## Discussion

STAT3 signaling plays a crucial role in tumorigenesis. In particular, JAK2 is important for cytokine receptor signal transduction [41,42]. After activation, JAK2 kinase can phosphorylate STAT3, which then translocates to the nucleus to enhance the levels of downstream genes, including anti-apoptotic genes that are implicated in human cancer development [43–45]. Blocking upstream activators of STAT3 or suppressing phosphorylation of STAT3 itself has been considered to be a potential anticancer strategy [46–49]. A number of natural products have been reported to inhibit STAT3 activity and induce apoptosis in different tumor cell lines [10,50,51]. However, to our knowedge, few natural products act via JAK2 inhibition or have been investigated for the potential treatment of malignant melanoma [10,52]. WP1066, a small-molecule, directly process of tumorgenesis and metastasis by inhibiting the phosphorylation of JAK2/STAT3 pathway and the subsequent downstream protein, such as survivin and c-Myc, which are associated with tumor formation [53]. BBMD3, another JAK2 inhibitor, which inhibited autophosphorylation of JAK2 and blocked activation of downstream STAT3 signaling in melanoma cells [10]. In our previous research, we identified amentoflavone and its analogues as inhibitors of JAK2 activity with antiproliferative activity against the leukemic HEL cell line [34]. Due to the prevalance and high mortality rate of metatsatic melanoma, we were interested to investigate the efficacy of amentoflavone and its analogues against this type of cancer. In this study, we found that the amentoflavone analogue **1** showed more potent antiproliferative activity (0.36 μM) against melanoma cells compared to other cancer cell lines such as PC3, DU145, A2058 and HepG2 cells (Fig 2A–2E). Moreover, compound **1** had no discernible toxicity on normal LO2 cells (Fig 2F). This suggested that compound **1** could potentially be developed as a selective antitumor agent against malignant melanoma.

The mechanism of action of compound **1** was evaluated using multiple cellular assays. Compound **1** reduced the phosphorylation of JAK2 and STAT3 in A375 melanoma cells, but had no discernible effect on total JAK2 and STAT3 levels (Fig 3). The cellular thermal shift assay (CETSA) has been applied as the validation and optimization of drug target engagement [39]. An obvious shift of the melting temperature of JAK2 protein was detected (Fig 4), indicating that **1** could engage JAK2 even in the presence of cellular debris.

In addition, the phosphorylation of JAK2 and STAT3 can enhance the levels of the anti-apoptotic proteins Bcl-xl, Bcl-2, MCL-1 and survivin [54–56]. Therefore, we investigated whether compound **1** could induce apoptosis via acting through the JAK2/STAT3 pathway. Our results showed that compound **1** could induce apoptosis in approximately 80% of A375 cells after 24 h. Compound **1** could also induce cleavage of PARP and decrease caspase-3 levels, which could account for the pro-apoptotic activity of the compound (Fig 5). Subsequently, we studied whether compound **1** down-regulates the antiapoptotic protein Bcl-2 level which is regulated by STAT3 [54]. Our results showed that compound **1** inhibited the level of Bcl-2 in melanoma cells (Fig 6). This is consistent with other reports showing that inhibition of constitutively activated STAT3 correlates with altered Bcl-2/Bax levels [57]. HIF1α and ubiquitin are associated with apoptosis [58–60]. In order to rule out compound **1** acting via HIF1α or ubiquitin-associated pathways to induce apoptosis, the levels of HIF1α and ubiquitin were measured in treated cells. The results showed that **1** has no discernible effect on HIF1α and ubiquitin levels, suggesting that it did not induce apoptosis via those HIF1α and protein degradation pathways (Fig 7).

Taken together, these results clearly suggest that the apoptotic effect of compound **1** in melanoma cells is due to inhibition of JAK2/STAT3 signaling leading to the activation of pro-apopotic regulators and the inhibition of anti-apoptotic regulators, rather than through a general toxicity mechanism. To the best of our knowledge, this study is the first to demonstrate use of an amentoflavone analogue as a JAK2 inhibitor with pro-apoptotic and anti-proliferative effects against melanoma cells.
